# Evaluating the efficacy of an online depression screening tool in South Africa: A pilot study

**DOI:** 10.4102/sajpsychiatry.v28i0.1687

**Published:** 2022-02-28

**Authors:** Tasneem Hassem

**Affiliations:** 1 Department of Psychology, Faculty of Human and Community Development, University of the Witwatersrand, Johannesburg, South Africa

**Keywords:** criterion validity, depression, online screening, reliability, sensitivity, specificity

## Abstract

**Background:**

A global increase of 16% in depression rates from 1990 to 2019 highlights the alarming situation in relation to increase in depression. Research has indicated that this rate is likely to increase as a result of the coronavirus disease 2019 (COVID-19) pandemic. In South Africa, the depression life-time prevalence rate is 9.47%. However, the lack of access to mental healthcare services leads to people not receiving much needed information and care. The growing accessibility to the Internet for South Africans offers a solution for the screening and access to self-help information for depression. The Center for Epidemiologic Studies Depression Scale (CESD)-R was adapted for online usage and a website, mddsa.co.za, was piloted in this regard.

**Aim:**

This study reports on the efficacy of the online adapted CESD-R for use in South Africa by reporting on the reliability and criterion validity as well as the user friendliness of the website and the appropriateness of the instant feedback provided.

**Setting:**

The study was conducted in South Africa during COVID lockdown level 1 and 2.

**Methods:**

This study followed a quantitative, cross-sectional research design. A convenience sample of 21 individuals, above the age of 18, with a depression diagnosis and 86 individuals with no mental health diagnosis participated in the study. Participants accessed the screening instrument online at the website.

**Results:**

Internal consistency reliability coefficients exceeded 0.80. T-test and sensitivity and specificity results attested to the accuracy of the tool. All items contributed well to the instrument, including the items that were culturally specific to South Africa. Feedback from participants indicated that the tool was easily comprehensible, the website was user friendly and the instant feedback provided was appropriate.

**Conclusion:**

The online adapted CESD-R evidenced excellent reliability and criterion validity and was able to accurately screen for depression amongst South Africans. The website and the tool have the potential to be utilised to increase access to a screening instrument for individuals who display symptoms of depression and to enhance the opportunity for individuals to practise self-help.

## Introduction

Depression is currently ranked as the 13th leading cause of global burden of Disability Adjusted Life Years (DAYLS) in 2019,^1^ as a result of a 16% increase in the global prevalence rate from 1990 to 2019. The life-time prevalence rate of depression in South Africa is 9.47%.^2^ Research suggests that depression rates are likely to increase as a result of the coronavirus disease 2019 (COVID-19) pandemic.^3,4^ This can be attributed primarily to experiences of isolation as well as other dramatic changes in social and occupational spheres during the pandemic.^5^

Research on depression in the South African context highlights unique symptoms experienced by individuals diagnosed with depression such as feelings of loneliness, not feeling like oneself, ‘thinking too much’ as well as an increased emphasis placed on somatic symptoms experienced and reported by individuals.^6,7,10,11^ These symptoms have not been included in the Diagnostic and Statistical Manual diagnostic criteria for depression, which states an individual needs to display at least one symptom of either being depressed or loss of interest of pleasure for more than two weeks and an additional five to nine symptoms present nearly every day.^12^ In South Africa the diagnosis and treatment of depression has been compromised for various reasons, such as, the challenges experienced in accessing mental healthcare, lack of mental health resources, depression terminology is often not available in all South African languages to describe the diagnosis, the term depression is not understood in the same way across cultures and the stigma associated with mental illnesses.^6,7,8,9^ An additional factor which compromises and often results in the underdiagnosis of depression is the instrument used to screen for depression amongst individuals.

Instruments which screen for depression are an additional source of information to assist with diagnosis and are mostly self-report instruments developed for Westernised countries, thus posing a variety of challenges which impact on the accuracy of these instruments. These instruments utilise psychological jargon when assessing symptoms which is not often understood by second language English speakers and translations of these instruments into indigenous South African languages often results in construed meaning of the constructs measured. In addition, there is an emphasis placed on assessing cognitive symptoms of depression and these instruments do not account for the unique depression symptoms identified in the South African context. Despite the unique presentation of depression experienced by South Africans, commonly used depression screening tools in the South African context have not been adapted, however they have been translated into various South African languages.^13,14,15^

The Center for Epidemiologic Studies Depression Scale (CESD) is amongst one of the commonly utilised screening instruments for individuals who have symptoms of depression that has been translated into three South African languages (Afrikaans, isiZulu and isiXhosa).^13^ The translated tool evidenced reliability scores ranging between 0.69 and 0.89, sensitivity and specificity ranging between 71.4% and 84.1% and 72.6% – 95%, respectively. Positive predictive values (PPV) ranged from 16.1% to 54.8%.^13^ The Centre for Epidemiological Studies Depression – Revised scale (CESD-R) administered on an electronic device (hand-held tablet) evidenced an internal consistency reliability score of 0.95, a sensitivity of 0.81 and specificity of 0.82 in a sample of HIV-positive South African individuals.^16^ A pooled analysis of the CESD has evidenced sensitivity and specificity of 87% (95% confidence interval [CI] 0.82–0.91) and 70% (95% CI 0.65–0.75), respectively, in a sample of general and primary care population.^17^ Internationally, the paper versions of the CESD, CESD-10 (10 items) and CESD-R evidenced reliability coefficients ranging from 0.94 to 0.83,^18,19,20,21,22^ while a Cronbach alpha of 0.82 was established for a shortened online version of the CESD (7-items) amongst college students in Spain.^23^

On the basis of the unique symptom presentation of depression, lack of mental health resources and the fact that 64.7% of South Africans have at least one member in their household who has access to the Internet and only 8.4% of individuals speak English as a home-language,^24^ [Author(s), in press] (under review) adapted the CESD-R for online usage within the South African context.^25^

The online depression screening tool is located on MDDSA.co.za, as an open access resource. The website provides the user with information regarding depression, the screening tool as well as various contact details for individuals who are in need of support. Once individuals take the test, they receive instant feedback regarding their risk level (low, medium and high) in terms of the depression symptoms they are experiencing. The online adapted CESD-R demonstrates good content validity^25^ and relevance, and a high internal consistent reliability of 0.93 amongst postgraduate university students. The efficacy of the tool for the general South African population has not been determined. Thus, this study investigated the reliability, criterion validity (sensitivity and specificity), comprehensibility and user friendliness of the online adapted CESD-R as well as the user friendliness of the website and appropriateness of the instant feedback provided.

## Methods

### Study design

The study followed a non-experimental, quantitative, cross-sectional research design, as participants completed a survey via the website (MDDSA.co.za). A request made for participation in the study was circulated by psychologists, psychiatrists and general practitioners on various social media platforms and in their consulting rooms. Data collection commenced on 28 September 2020 and closed on 30 November 2020. It should be noted that data collection occurred during the COVID lockdown Levels 1 and 2 in South Africa. During lockdown Levels 1 and 2, all individuals were required to wear face masks when in public places and all major sectors were permitted to resume operations. Access to hospitals were only permitted for obtaining medication and seeking treatment, while adhering to strict health protocols.^26^

### Study population

A non-probability convenience sample of 107 individuals participated in the study.^27^ Table 1 highlights the sample demographics. The majority of the sample (*n* = 86) were not diagnosed with depression (No diagnosis [ND] sample), whereas 21 individuals reported having received a formal depression diagnosis (formally diagnosed [FD]). The majority of the ND sample identified as being female (*n* = 60, 69.8%), black people (*n* = 25, 37.9%), Christian (*n* = 40, 46.5%), and spoke English as their home language (*n* = 50, 58.1%). The FD were mainly female (*n* = 13, 61.9%), white people (*n* = 50.0%), Christian (*n* = 42.9%) and spoke English (*n* = 17, 81%). The ND participants had an age range of 19–70 years old (M = 35, SD = 12.205), while the age range for the FD participants was 19–66 (M = 33.5, SD = 11.405).

**TABLE 1:** Combined, No diagnosis and formally diagnosed sample demographics.

Demographics	Variables	Combined sample		ND sample		FD sample	
		Frequency	Percentage	Frequency	Percentage	Frequency	Percentage
Gender	Female	73	68.2	60	69.8	13	61.9
	Male	34	31.8	26	30.2	8	38.1
Race†	Black	28	35	25	37.9	3	21.4
	Coloured	8	10	6	9.1	2	14.3
	Indian	15	18.8	14	21.2	1	7.1
	White	25	31.3	18	27.3	7	50
	Asian	2	2.5	1	1.5	1	7.1
	Other	2	2.5	2	3	2	14.3
Religious affiliation	Christianity	49	45.8	40	46.5	9	42.9
	Hinduism	9	8.4	6	7.0	3	14.3
	Islam	27	25.2	25	29.1	2	9.5
	Judaism	6	5.6	5	5.8	1	4.8
	No religious affiliation	11	10.3	7	8.1	4	19.0
	Traditional African	3	2.8	3	3.5	-	-
	Other	2	1.9	-	-	2	9.5
Home Language	Afrikaans	5	4.7	5	5.8	-	-
	English	67	62.6	50	58.1	17	81.0
	Sepedi	4	3.7	4	4.7	-	-
	Setswana	12	11.2	10	11.6	2	9.5
	Sotho	4	3.7	2	2.3	2	9.5
	Tshivenda	2	1.9	2	2.3	-	-
	Xitsonga	3	2.8	3	3.5	-	-
	isiXhosa	2	1.9	2	2.3	-	-
	isiZulu	4	3.7	4	4.7	-	-
	Non-South African	4	3.7	4	4.7	-	-
Language proficiency (ability to speak and undertake various tasks)	Excellent	79	73.8	62	72.1	17	81.0
	Good	25	23.4	21	24.4	4	19.0
	Poor	2	1.9	2	2.3	-	-
	Very poor	1	0.9	1	1.2	-	-
Language comprehension (ability to understand)	Excellent	80	74.8	63	73.3	17	81.0
	Good	26	24.3	22	25.6	4	19.0
	Very poor	1	0.9	1	1.2	-	-
Reading skills	Excellent	83	77.6	66	76.7	17	81.0
	Good	22	20.6	18	20.9	4	19.0
	Poor	1	0.9	1	1.2	-	-
	Very poor	1	0.9	1	1.2	-	-
Have you been diagnosed with a physical illness	No	84	78.5	71	17.4	13	61.9
	Yes	23	21.5	15	82.6	8	38.1
Are you currently taking medication for your illness	No	79	73.8	70	81.4	9	42.9
	Yes	28	26.2	16	18.6	12	57.1
Have you been diagnosed with depression previously	No	86	80.4	86	100	-	-
	Yes	21	19.6	-	-	21	100

Note: *N* = 101, except where indicated otherwise,

† *N* = 80.

ND, No diagnosis; FD, formally diagnosed.

In the ND sample, nine of the 11 official languages of South Africa were selected as a home language, whereas only three of the 11 languages were selected as the home language by the FD sample. With regards to comprehension and reading ability in English, majority of both the ND and FD samples rated their ability as excellent. Majority of the ND and FD samples reported not having been diagnosed with a physical chronic condition (see Table 1).

The majority of the FD sample reported being diagnosed with depression by a psychiatrist (*n* = 17, 81%) and stated that they had a depressive episode at least within the past 6 months of taking the survey (*n* = 12, 57.1%). In the FD sample, 12 out of the 21 individuals were on medication to treat their depression as is evident in Table 2.

**TABLE 2:** Depression History of the depressed sample.

Depression history	Variable	Frequency	Percentage
**Who diagnosed you with depression?**	General Doctor	4	19
	Psychiatrist	17	81
**Last depression episode**	A year ago	9	42.9
	During this month	4	19
	In the past two months	7	33.3
	In the past six months	1	4.8

Note: *N* = 21

### Instruments

The survey consisted of a brief demographic questionnaire, the adapted online CESD-R as well as several questions assessing the comprehensibility and user friendliness of the online adapted CESD-R, the user friendliness of the website and the appropriateness of the instant feedback provided. The brief demographic questionnaire requested information regarding age, gender, population group, religious affiliation, home language, health condition, depression diagnosis. Participants who answered ‘Yes’ to being diagnosed with depression had three follow-up questions relating to year of diagnosis, who made the diagnosis as well as the occurrence of the last depressive episode. Lastly, participants were asked to rate ability in English from e*xcellent* to *very poor* with regards to proficiency, comprehension and reading ability.

The online adapted CESD-R is grounded in the Biopsychosocial-Spiritual (BPSS) model and consists of 19 items with a 4-point Likert type response format (0 = Not at all, 1 = Some of the time, 2 = Most of the time and 3 = All the time). It assesses symptoms over a two-month period. In addition, the items are jargon free and can be easily understood. Four items pertain specifically to the idioms of distress experienced by the South African population, namely, ‘I have been experiencing more body aches and pains (e.g. headaches, neck pain or back pain)’, ‘I have been thinking too much’, ‘I have been feeling alone’ and ‘I have not felt like myself’. The tool is scored out of 57 and uses a two-tier scoring system. Tier one looked at symptoms of sadness and loss of interest, while Tier 2 focussed on appetite, sleep, concentration, guilt, fatigue and movement based on the symptom presentation outlined in the DSM-5.^28^ A cut-off score of 20 and less placed individuals into the low-risk category, a score ranging from 21 to 34 placed individuals in a medium-risk category and a cut-off score of 35 and above placed individuals in high-risk category. The tool displayed good content validity in the South African context.^24^

Lastly, participants were asked to indicate via a Yes/No response format on the user-friendliness of the tool and the website, if the instructions provided were easily understood, item appropriateness as well as to indicate if there were any words or phrases they did not understand. After completion, participants were presented with the results of the CESD-R and asked to comment on the appropriateness of the feedback provided.

### Procedure

Participants received information about the study via psychologists, psychiatrists, general practitioners and through social media such as WhatsApp. Information about the study included a link to the survey on the MDDSA.co.za website. The survey took approximately 15 min to complete and participants were provided with instant results based on their item responses on the online adapted CESD-R.

### Ethical considerations

Approval to conduct the study was obtained from the Human Research Ethics Committee – Medical (HRECM) of the University of the Witwatersrand, reference number: M180402. Participation in the study was completely voluntary and anonymous. Participants were informed about the study via a participant information sheet and free online and telephonic counselling details were provided to participants in the event of experiencing any form of distress.

### Data analysis

Data was extracted from the website database and coded for analysis. IBM Statistical Package for the Social Sciences (SPSS) Statistics 27 and JASP was used to analyse the coded data. Demographic variables as well as the six questions regarding the tool and website were analysed using frequencies and percentages. In order to determine the internal consistency reliability a Cronbach’s alpha coefficient and the McDonald’s Omega coefficient was calculated. To determine the criterion validity (sensitivity, specificity, PPV and negative predictive values [NPV]) of the tool were calculated are per the recommendations made by Trevethan.^29^ The Area Under the Receiver Operating Characteristic Curve was used to determine the accuracy of the tool. All the items were normally distributed as per skewness calculations. In order to determine the discriminatory power of the items amongst the ND and FD samples, an independent samples *t*-test was utilised; and where results were significant the Cohen’s d was calculated to determine the effect size.

## Results

### Descriptive statistics

Table 3 highlights the means scores obtained for both the ND and FD samples. For all items, the mean scores for the FD sample were larger than the mean scores for the ND sample; however, all differences were statistically significant (*p* < 0.05) with the exception of items 2, 3, 6 and 7 (*p* > 0.05). Large effect sizes ranging between 0.906 and 1.021, was evident for items 1, 5, 8, 9, 10, 11, 12 and 18, while moderate effect sizes ranging between 0.785 and 0.888 was evident for items 4, 13, 14, 15, 16, 17 and 19. Lastly, the mean total score for the FD sample was significantly higher than the mean total score for the ND sample (t_105_ = 4.22, *p* = 0.000; Cohen’s d = 12.239).

**TABLE 3:** Descriptive statistics and independent samples t-test.

Item	Combined sample†		ND sample‡		FD sample§		Cronbach alpha if item is deleted	Independent samples t-test		
	Mean	Standard Deviation	Mean	Standard Deviation	Mean	Standard Deviation		*t*	*P*-value	Cohen’s d
1. I have been experiencing more body aches and pains (e.g. headache, neck pain or back pain)	1.26	0.94	1.14	0.88	1.76	1.00	0.953	2.83	**0.006** ***	0.906
2. I have been thinking too much	1.70	0,91	1.64	0.94	1.95	0.74	0.950	1.64	0.110	0.909
3. I have been feeling sad or down	1.14	0.77	1.08	0.77	1.38	0.74	0.950	1.61	0.110	0.765
4. I had trouble keeping my mind on what I was doing	1.07	0.84	0.92	0.76	1.71	0.90	0.950	4.16	**0.000** ***	0.785
5. My weight has changed without me trying (lost weight or gained weight)	1.07	1.04	0.93	0.99	1.67	1.07	0.951	3.01	**0.003** ***	1.006
6. I felt like I have been moving too slowly	1.04	0.92	0.98	0.84	1.29	1.19	0.951	1.12	0.272	0.917
7. I could not make a decision about simple things	0.79	0.95	0.70	0.93	1.14	0.96	0.952	1.95	0.054	0.940
8. I could not get rid of this sad feeling	0.97	0.94	0.85	0.91	1.48	0.87	0.949	2.84	**0.005** ***	0.906
9. I have lost interest in my usual activities	1.00	0.98	0.81	0.88	1.76	1.04	0.948	4.28	**0.000** ***	0.909
10. I felt that most things are my fault	1.16	1.05	1.03	1.35	1.00	1.11	0.949	2.54	**0.013** ***	1.021
11. I have not liked myself	0.93	1.02	2.15	0.71	0.92	0.93	0.950	4.91	**0.000** ***	0.921
12. My sleep has changed (having trouble sleeping or sleeping more than usual)	1.35	1.06	2.63	1.16	0.99	1.00	0.950	3.86	**0.000** ***	0.993
13. I could not do things that I’ve always done	0.86	0.90	2.43	0.76	0.83	1.06	0.950	2.49	**0.014** ***	0.874
14. I have been feeling tired	1.45	0.92	2.99	1.27	0.87	0.87	0.949	4.46	**0.000** ***	0.851
15. I could not focus on important things	0.98	0.89	2.69	0.83	0.77	1.07	0.949	3.90	**0.004** ***	0.836
16. My eating has changed (eating less than normal/more than normal)	1.07	0.94	2.62	0.90	0.85	1.00	0.950	4.03	**0.000** ***	0.883
17. Nothing has made me happy	0.82	0.92	2.26	0.70	0.84	1.07	0.949	2.94	**0.004** ***	0.888
18. I have been feeling alone	1.09	1.01	2.52	0.99	1.01	0.93	0.950	2.21	**0.029** ***	0.996
19. I have not felt like myself	1.06	0.90	2.70	0.95	0.87	0.93	0.949	2.44	**0.016** ***	0.879
Total depression score	20.80	13.17	18.34	12.16	30.90	12.57	-	4.22	**0.000** ***	12.239

Note:

*** Significant at α = 0.05,

† *N* = 107,

‡ *N* = 86,

§ *N* = 21.

ND, No diagnosis; FD, formally diagnosed.

### Reliability of the adapted online Center for Epidemiologic Studies Depression Scale-R

As is evident in Table 4, the online adapted CESD-R displays an excellent internal consistency reliability with a Cronbach’s alpha coefficient of 0.952 and McDonald’s omega coefficient of 0.954 for the combined samples.^30^ The Cronbach alpha for the FD sample was 0.934, while the McDonald omega was 0.938. For the ND sample, the Cronbach alpha coefficient was 0.948 and the McDonald omega coefficient was 0.950. Table 4 also demonstrates the effect on reliability if an item is excluded. There are no significant increases or decreases to the reliability coefficients if any of the items are excluded. Thus, each item contributes well to the tool.

**TABLE 4:** Reliability analyses.

Item	Cronbach alpha if item is deleted	McDonalds Omega if item deleted
I have been experiencing more body aches and pains (e.g. headache, neck pain or back pain)	0.953	0.954
I have been thinking too much	0.950	0.951
I have been feeling sad or down	0.950	0.951
I had trouble keeping my mind on what I was doing	0.950	0.951
My weight has changed without me trying (lost weight or gained weight)	0.951	0.952
I felt like I have been moving too slowly	0.951	0.952
I could not make a decision about simple things	0.952	0.953
I could not get rid of this sad feeling	0.949	0.950
I have lost interest in my usual activities	0.948	0.950
I felt that most things are my fault	0.949	0.951
I have not liked myself	0.950	0.951
My sleep has changed (having trouble sleeping or sleeping more than usual)	0.950	0.952
I could not do things that I’ve always done	0.950	0.951
I have been feeling tired	0.949	0.950
I could not focus on important things	0.949	0.950
My eating has changed (eating less than normal/more than normal)	0.950	0.951
Nothing has made me happy	0.949	0.951
I have been feeling alone	0.950	0.952
I have not felt like myself	0.949	0.950
Combined sample	0.952	0.954
FD sample	0.934	0.938
ND sample	0.948	0.950

ND, No diagnosis; FD, formally diagnosed.

### Validity of the adapted online Center for Epidemiologic Studies Depression Scale-R

As shown in Table 5, the majority (*n* = 45, 52.37%) of the group without depressive features scores ranked them in the low-risk category in the ND sample, while in the FD sample the majority of the participants score ranked them in the high-risk category (*n* = 10, 47.6%). In order to determine the sensitivity, specificity, PPV and NPV, medium- and high-risk group were combined to represent participants who display depressive features. In addition, reporting being FD with depression constituted the ‘Gold Standard’. Therefore, for the ND sample 41 participants (47.7%) were classified as displaying depressive symptoms, while in the FD sample 19 (90.5%) participants were classified as displaying prominent depressive features (Table 6).

**TABLE 5:** Depression symptom risk category for the combined, No diagnosis and FD samples.

Depression risk category	Combined sample		ND Sample		FD sample	
	Frequency	Percentage	Frequency	Percentage	Frequency	Percentage
Low risk	47	43.9	45	52.3	2	9.5
Medium risk	30	28	21	24.4	9	42.9
High risk	30	28	20	23.3	10	47.6

ND, No diagnosis; FD, formally diagnosed.

**TABLE 6:** Basis for deriving sensitivity, specificity, positive and negative predictive values.

Variable	Result
True positive	*N* = 19
False negative	*N* = 2
False positive	*N* = 41
True negative	*N* = 45

**FIGURE 1:**
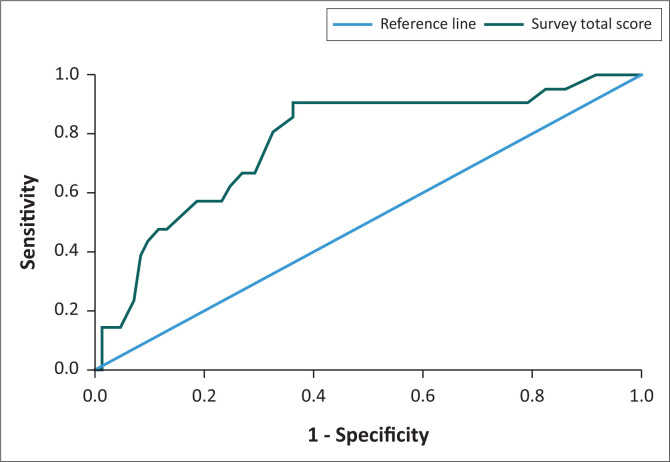
ROC Curve showing the AUC for the online adapted CESD-R.

With a cut-off score of 20, the tool produced a sensitivity of 90.48% and a specificity of 47.67%, while the positive predictive value was 31.67% and negative predictive value was 95.75%. When looking at the ROC curve, it is evident that the test has a fair accuracy with AUC (area under the curve) equal to 0.776 and the accuracy of the tool is statistically significant at a 95% confidence interval (*p* = 0.000; 0.6631–0.889) (see Figure 1).

As evidenced in Table 7, the website and depression screening tool were viewed as user friendly by the majority of participants (*n* = 103, 98.1%). The majority of the participants reported that the instructions of the tool were easily understood (*n* = 104, 99%). Participants noted that terminology used to define symptoms were easy to understand (*n* = 101, 97.1%) and reported that the items or phrases in the tool were appropriate (*n* = 100, 96.2%). With regards to the instant feedback provided only 90 participants responded, with majority (*n* = 86, 96%) indicating the feedback provided was useful.

**TABLE 7:** Tool and website feedback for combined, ND and FD samples.

Variable	Yes/No	Combined		ND sample		FD sample	
		Frequency	Percentage	Frequency	Percentage	Frequency	Percentage
Is the website user friendly?†	No	2	1.9	1	1.2	1	4.8
	Yes	103	98.1	83	98.8	20	95.2
Is the tool user friendly?‡	No	1	0.98	1	1.2	-	-
	yes	102	99	81	98.8	21	100
Were the instructions easily understood?§	No	1	1.0	1	98.8	-	-
	yes	104	99.0	83	1.2	21	100
Any words or phrases that were not understood?¶	No	101	97.1	81	97.6	20	95.2
	Yes	3	2.9	2	2.4	1	4.8
Were the items appropriate?††	No	4	3.8	3	3.6	1	4.8
	yes	100	96.2	80	96.4	20	95.2

Note:

†,§ *N* = 105,

‡ *N* = 103,

¶,†† *N* = 104.

ND, No diagnosis; FD, formally diagnosed.

## Discussion

This study set out to assess the reliability, criterion validity (sensitivity and specificity) comprehensibility as well as the user friendliness of the online adapted CESD-R tool. In addition, the user friendliness of the website as well as the appropriateness of the instant feedback provided was assessed. Results indicate that the online adapted CESD-R is reliable, valid, user friendly and comprehensible. In addition, the website on which the tool is located is user friendly and the instant feedback provided is appropriate.

The 19 items on the tool are each able to discriminate between individuals who present with depressive features and individuals who do not display prominent symptoms of depression, as the FD sample obtained statistically higher means on 15 (1, 4, 5, 8–19) out of the 19 items when compared to the ND sample. Items that did not display a statistical difference in mean scores between the FD and ND sample, assessed concentration (3, 7), sadness (2) as well as movement (6).

The four items which constitute symptoms unique to individuals who are diagnosed with depression in South Africa, which are not included in the diagnostic manual used for classifying and diagnosing depression can be deemed appropriate. The appropriateness of these items (1, 18, 19) is reflective in a statistically higher mean obtained by the FD sample when compared to the ND sample. In addition, these items all contribute to the overall reliability score of the tool and removal of any of these items does not increase the overall reliability score of the tool.

The online adapted CESD-R evidenced an excellent reliability scores, which is higher than the lower and equivalent upper range of the paper-based CESD-10^13^ and equivalent to the CESD-R administered on a hand-held tablet within the South African context.^16^ When compared to the reliability scores established on the paper-based version of the CESD, CESD-10 and the CESD-R, the adapted version evidenced a higher reliability coefficient.^18,19,20,21,22^ Lastly, the online adapted CESD-R displays a higher reliability coefficient when compared to the online CESD administered to a Spanish college sample^23^ and the online adapted CESD-R administered to a South African postgraduate sample.

The online adapted CESD-R displayed a higher sensitivity and a lower specificity score in relation to the paper-based CESD-10 and CESD-R administered on a hand-held tablet within the South African context.^12^ The higher sensitivity score can be attributed to the easy-to-understand language, the inclusion of the symptoms displayed by South Africans FD with depression as well as the removal of positive affect items which performed poorly on the paper-based CESD-10.^13^ The lower specificity score can be attributed to the timing of the study, where depression is viewed as a psychological reaction to the COVID-19 pandemic,^5^ thus, increasing depression symptoms experienced by the ND sample. The low PPV and high NPV evidenced is in accordance with that reported by Baron et al.^13^ However, the PVV is lower and the NPV is higher than those reported by Kagee et al.,^16^ which can be attributed to the higher prevalence rate of depression amongst the sample recruited by Kagee et al.^16^ The low PPV is a direct result of the relatively small sample size of FD depressed individuals in the study.

As a result of the removal of psychological jargon from the online adapted tool, it is evident that the instructions as well as items can be easily understood by individuals who are not first language English speakers. The user friendliness of the tool and the website highlights the potential the tool has in allowing individuals to assess their symptoms in the comfort of their own homes and on their own time, thus holding the potential to reduce the stigma associated with depression within the community settings. Lastly, the instant feedback provided to all risk groups (low, medium, and high) was well received, thus highlighting the appropriateness of the way feedback is displayed.

## Limitations

The sample size and the time at which the study was conducted are limitations in this study. As a result of the COVID-19 pandemic and limited access to hospitals and treatment facilities, the researcher was not able to obtain a larger and more representative sample of individuals diagnosed with depression. In addition, many individuals with no history of depression may have experienced symptoms of depression as a result of the effects of the pandemic. As a result of the sample size, more sophisticated statistical techniques such as item response theory analysis and confirmatory factor analysis could not be performed. Therefore, it is recommended that testing continue to obtain a larger and more representative sample size.

## Conclusion

The study provides evidence that the online adapted CESD-R displays good reliability and validity while accounting for the unique symptoms of depression experienced by South Africans. As a result of the ease of accessibility and user friendliness of the tool, the adapted online CESD-R has the potential to be utilised in both public and private healthcare facilities in South Africa as an adjunct to the clinical observations that are usually done on the clinical setting. Lastly, the instant feedback provided as well as the information on self-help and contact details for further assistance can be viewed as a step towards the creation of awareness of the symptoms of distress that might lead to a diagnosis of depression and it might assist individuals to seek more formal modes of assessment and treatment if necessary.
